# Early childhood developmental status and its associated factors in Bangladesh: a comparison of two consecutive nationally representative surveys

**DOI:** 10.1186/s12889-023-15617-8

**Published:** 2023-04-12

**Authors:** Mohammad Nayeem Hasan, Md. Rashed Babu, Muhammad Abdul Baker Chowdhury, Mohammad Meshbahur Rahman, Nafiul Hasan, Russell Kabir, Md Jamal Uddin

**Affiliations:** 1 Department of Statistics, Shahjalal University of Science and Technology, 3114 Sylhet, Bangladesh; 2 Department of Neurosurgery, University of Florida College of Medicine, Gainesville, FL USA; 3 National Institute of Preventive and Social Medicine, 1212 Mohakhali, Dhaka, Bangladesh; 4 School of Allied Health, Anglia Ruskin University, Essex, UK; 5 Department of General Educational Development (GED), Daffodil International University, Dhaka, Bangladesh

**Keywords:** ECD, Child literacy-numeracy development, Child physical domain, Social-emotional development, Approaches to learning domain, Multiple indicator cluster surveys (MICS), Bangladesh

## Abstract

**Background:**

Inadequate cognitive and socio-emotional development in children leads to physical and mental illness. We aimed to investigate the status of early childhood development (ECD) and its associated factors. Additionally, aimed to compare the changes of significantly associated factors using two multiple indicator cluster surveys (MICS) in Bangladesh.

**Methods:**

We used data from the Multiple Indicator Cluster Surveys (MICS) 2012 and 2019 nationally representative surveys. A total of 17,494 children aged 36–59 months were included in the analysis. The outcome variable was ECD status: either developmentally on-track or not. We used bivariable analysis and crude and adjusted multivariable logistic models to assess the ECD status and its associated factors.

**Results:**

Comparing both MICS surveys, the overall and individual domains of ECD status improved from 2012 (65.46%) to 2019 (74.86%), and the indicators of child literacy-numeracy domain improved from 21.2 to 28.8%, physical domain improved from 92.2 to 98.4%, and social-emotional domain improved from 68.4 to 72.7%. The learning approach domain was 87.5% in 2012 and increased to 91.4% in 2019. According to the adjusted logistic model in both surveys (2012 and 2019), the age of 4 years had an adjusted odds ratio (AOR) of 1.61 and 1.78 times higher developmentally on track than the age of 3. Female children were 1.42 (in 2012) and 1.44 (in 2019) times more developmentally on track than males. Compared to mothers with only primary education, children raised by mothers with secondary or higher education were 1.77 and 1.50 times more on track in their development. Moreover, Children from affluent families had 1.32- and 1.26 times higher odds- on track than those from the poorest families. Families with books had 1.50 and 1.53 times higher developmentally on track than their counterparts.

**Conclusion and recommendation:**

In summary, our study shows that the overall ECD status improved between MICS 2012 and MICS 2019. Important factors influence ECD status, including early childhood education programs, families’ possession of children’s books, mothers’ educational level, and wealth index. The findings of our study will help making necessary public health-related initiatives in Bangladesh to improve ECD program.

**Supplementary Information:**

The online version contains supplementary material available at 10.1186/s12889-023-15617-8.

## Background

A child’s cognitive development, social and emotional characteristics are all influenced by their formative years [1]. ECD relates to a child’s physical, mental, socio-emotional, and growth in motor skills during a child’s productive periods [2]. From the prenatal period through infancy and childhood, a child’s quickly expanding brain is incredibly prolific and proactive [3]. This is the golden period for them to make themselves highly thirsty for learning and physically fit to become a successful and productive person in later life [4]. Within five years of birth, children begin to learn about the world around them, and this evolution is linked to the development of physical, verbal, perceptual, and psychological changes [5]. This allows them to stay focused, understand and follow directions, communicate with others, and solve increasingly complex problems [1].

According to world bank press news in March 2021, more than 40% of children below primary-school age need childcare but don’t have access. Between 2010 and 2016, 25.3% of children in 63 low and middle-income countries (LMICs) had a developmental deficit, with 10.1% in Europe and Central Asia, 32.6% in South Asia, 17.0% in East Asia and Pacific, and 41.4% in West and Central Africa experiencing developmental delays [6]. For Sub-Saharan Africa (SSA), the median prevalence of cognitive 16.1% was not on track, and 28.6% of the social-emotional domain was not developmentally on track [7]. For Bangladesh, based on multiple indicator cluster surveys (MICS) 2012, 70% of the children were developmentally on track [8], and for Bangladesh MICS 2019, 25.26% of the children were not developmentally on track with ECDI [9].

ECD has become increasingly popular worldwide since the turn of the twenty-first century. According to developed countries, population-based measures may help measure ECD and predict later life wellness [1]. Yet, despite the practical importance of the ECD, population-based estimates have not been readily available in LMIC countries [10].

According to Rana et al., (2022), household air pollution from solid fuel use is linked to ECD [11]. Alam et al., (2021) investigated the current ECD status of young Bangladeshi children aged 3–4 years and how it relates to various sociodemographic and familial aspects [9]. Increased parental stimulation involvement benefits ECD in LMICs [12]. In 63 low-and middle-income countries, Gil et al., (2020) looked at the prevalence and inequality of putative delays in child development [6]. Kang et al., (2018) provided findings from a study examining the links between undernutrition and indices of learning/cognition and social-emotional development in South Asian children aged 36 to 59 months. In South Asia, stunted children become less developmentally on track in the learning and cognitive domains [13]. Islam et al., (2021) investigated the relationship between developmental status and various socio-demographic and environmental factors that could influence children’s development [14].

The Lancet 2016 child development series concluded, using data from UNICEF (United Nations International Children’s Emergency Fund) and the World Bank, that 43 per cent of children under five fail to achieve their developmental potential each year. Children in LMIC countries risk suboptimal development due to poverty, stunting, microbial shortages, contagious diseases, environmental exposure, and psychological issues [15], [16]. In Bangladesh, government and non-government organizations work with many developmental facilities for children, child parents, and caretakers to ensure all rights they deserve [17]. Creating an innovative foundation for strong development during the early years of life is essential for thriving communities, economic productivity, and civil societies. But most parents in Bangladesh are unaware of this scientific fact, which forms the core of ECD. UNICEF continues to promote the idea of ECD, show how policies work, strengthen networks and partnerships, and offer technical support and assistance [18] to people unaware of it. However, empirical research on overall ECD status and comparing different survey data is lacking. As a result, we sought to determine whether the ECD status and its associated factors changed in two consecutive Multiple Indicator Cluster Surveys (MICS) in Bangladesh.

## Methodology

We followed the STROBE guideline for better observational cross-sectional study reporting in epidemiology.

### Data source

We used two consecutive data from the Multiple Indicator Cluster Survey (MICS) conducted in 2012 and 2019. MICS, an intensive, multidimensional, nationally representative household survey, is administered by UNICEF. This study uses standardized questionnaires to gather data and critical indicators about children. This survey primarily focuses on reproductive women’s health, maternity and child health interventions, child nutrition, and early childhood development. MICS also collects an identical set of socioeconomic characteristics of individuals and households [19], [20]. Datasets were open access for the public domain [21].

### Sampling design and sample size

**Fig. 1:**
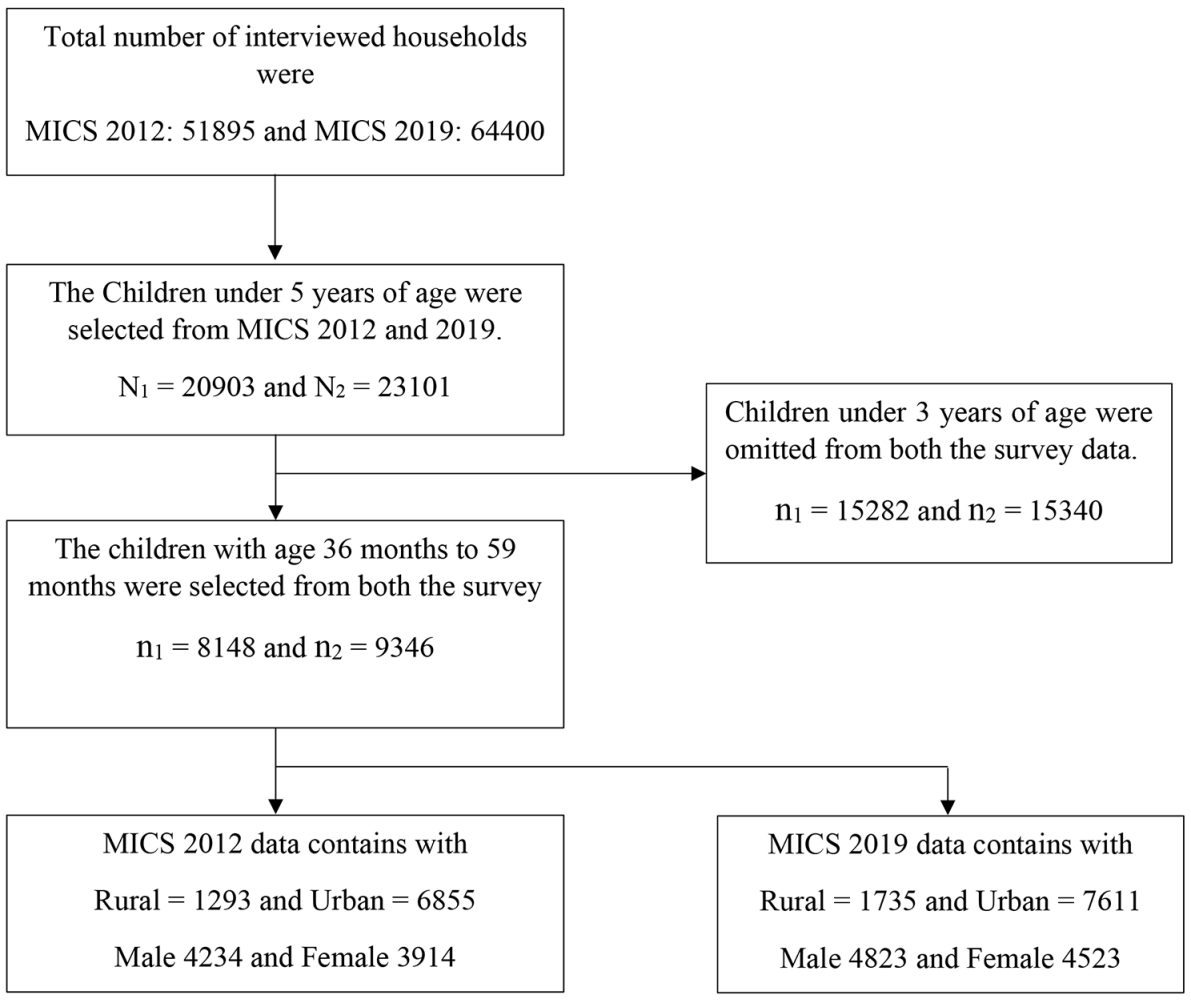
Schematic diagram of the analytic study sample

MICS survey is a double-stage cluster sampling procedure, randomly selecting households with children under five years. The 2012 MICS survey contained a sample of 51,895 households with a 51,116 (98.5%) response rate, while the 2019 MICS is based on a sample of 61,246 households with a 60,878 (99.4%) response rate. MICS gives an in-depth picture of children’s and women’s health in Bangladesh’s seven divisions (Dhaka, Chittagong, Sylhet, Rajshahi, Rangpur, Barisal, and Khulna). At stage two, districts were designated as the key sample strata for sample selection [19, 20]. In this study, the child age ranging from 36 to 59 months was selected. Therefore, this study included 17,494 children, where 8148 were in 2012 MICS and 9346 children in 2019 MICS having the information about the ECD and used in the analysis (*see* Fig. 1).

### Outcome variables

The Early Childhood Development Index (ECDI), developed by UNICEF, is a significant contribution. UNICEF’s ECDI was launched for the first time in 2009 during the fourth round of MICS (2012 MICS) and is now available in the following survey. The ECDI took shape when UNICEF, in collaboration with countries and partners, created measures to assess a child’s home environment and access to early childhood care and education. It contains ten dichotomous (yes/no) items in the categories of child literacy-numeracy development (3 items), child physical development (2 items), child social-emotional development (3 items), and child learning development (2 items) were four early developmental domains. The MICS includes questions from the ECD module for children under five and is aimed at mothers (or caregivers) of children aged three and four [10].

For creating our outcome variable (ECD status), we gave each child a score of 1 depending on the number of items to which the mother said yes, and a score of 0 otherwise. The ECD status variable was then constructed and used as the outcome variable based on the sum of these scores. This had a possible range of 0 to 4, with at least three of these four domains or scores greater than or equal to three indicating that the child was developmentally ‘on track’. The rest scores were treated as if they were developmentally ‘on delay’ [19, 20].

### Possible factors

For identifying the possible factors associated with ECD status, a set of independent factors such as the child’s age, sex, place of residence (urban vs. rural), geographical location (division), educational level of mothers (secondary complete or higher, secondary incomplete, primary complete, and primary incomplete), wealth index (richest, middle, and poorest) [22], religion (Islam and others), household head’s sex, household head’s ethnicity (Bengali and others), mother’s age, early childhood diseases, nutritional status (underweight, stunting, wasting, and overweight), early childhood educational program, mother stimulation, father stimulation, other stimulation, salt iodization, books, toys, sanitation facility (unimproved and improved) [23], access of media (television, newspaper or radio), and child punishment was used.

Some additional explanations for some variables are as follows: the WHO recommends using three anthropometric indices to assess a child’s nutritional status: height-for-age z-score (HAZ), weight-for-age z-score (WAZ), and weight-for-height z-score (WHZ) [2]. If the WHZ, HAZ, or WAZ was less than − 2, the child was classified as wasted, stunted, or underweight. A child was considered overweight if their WHZ was greater than + 2 [24]. Early childhood diseases were categorized into “yes” if the mother (or caretaker) of the child reported that the child had such symptoms (diarrhoea, symptoms of acute respiratory infection or fever); otherwise “no”. In this study, adults in the household were asked to participate in the following activities with children: reading books or looking at picture books with them, telling stories, singing songs, taking children outside the home, compound, or yard, playing with them, and spending time with them naming, counting, or drawing objects [19, 20]. We categorized “yes” if (fathers/mothers/others) have participated in any one activity with their children, otherwise “no”. Inadequate supervision is defined as a child under the age of five who has been left alone or under the supervision of another child under the age of ten for more than one hour at least once in the previous week [19, 20]. Salt iodization was categorised into “yes” if the iodine level was between 0 and 15 ppm or above 15 ppm and “no” if the iodine level was 0 ppm or no salt in the house [19, 20]. If a child aged 1 to 14 years had been subjected to physical or psychological abuse by caregivers in the previous month, they were considered to be subjected to child punishment [19, 20].

### Statistical analysis

First, a bivariable analysis was used to assess the relationship between ECD status and other factors. The univariable multi-level [unadjusted] and multivariable multi-level [adjusted] logistic regression analysis were performed separately for the 2012 and 2019 MICS survey data to compare the associated factors in these two periods. Multi-level logistic regression models take into consideration subject clustering inside clusters of higher-level units when determining the impact of subject and cluster characteristics on subject results [25]. In univariable analyses, one variable is added simultaneously in the multi-level logistic regression model (Table S1). For the adjusted model, all possible variables were added together in the model (Table 1). To account for the complicated survey design, we employed Stata’s Svyset tool (StataCorp LP, College Station, Texas). We employed sample weight in all analyses by design features like the primary sampling unit (PSU), stratum, cluster, and sample weight with the Svyset command [26].

**Table 1:** Comparison of the developmentally on-track status for indicated domains between two consecutive MICS survey

Domains	2012 MICS (%)	2019 MICS (%)
Literacy-numeracy	21.2	28.8
Physical	92.2	98.4
Social-Emotional	68.4	72.7
Approaches to learning	87.5	91.4

### Variable selection

Variables were selected in two stages. In the first stage, bivariable analysis (chi-square test) was conducted separately for each of the 26 variables. In total, 18 (MICS 2012) and 14 (MICS 2019) variables were selected by using a 5% level of significance for the adjusted logistic regression model (Table 1). The second stage created a comprehensive multivariable modelwith the selected predictor variables. With a cut-off value of 4.00, we also used the variance inflation factor (VIF) value to analyse multicollinearity in the final model [27]. All variables were included in the model in this stage because the VIF values of each variable were less than 4.00.

### Model performance

The Area under the Receiver Operating Characteristic (AUROC), sensitivity, and specificity parameters are used to assess the accuracy of the best model. Higher ROC areas suggested that the models performed better A lower P-value on the ROC curve shows that the model does differentiate between two groups, and the area under the ROC curve is bigger than 0.50 [28]. The intraclass correlation coefficient (ICC), cluster level variance with standard error, Akaike information criterion (AIC), Bayesian information criterion (BIC), and Log-likelihood were used to report the variation of ECD status at the community level and to test the model.

### Ethics statements

This freely available secondary data analysis was exempt from ethics assessment because no study on human subjects was done as part of this project.

## Results

### Socio-demographic characteristics

**Fig. 2:**
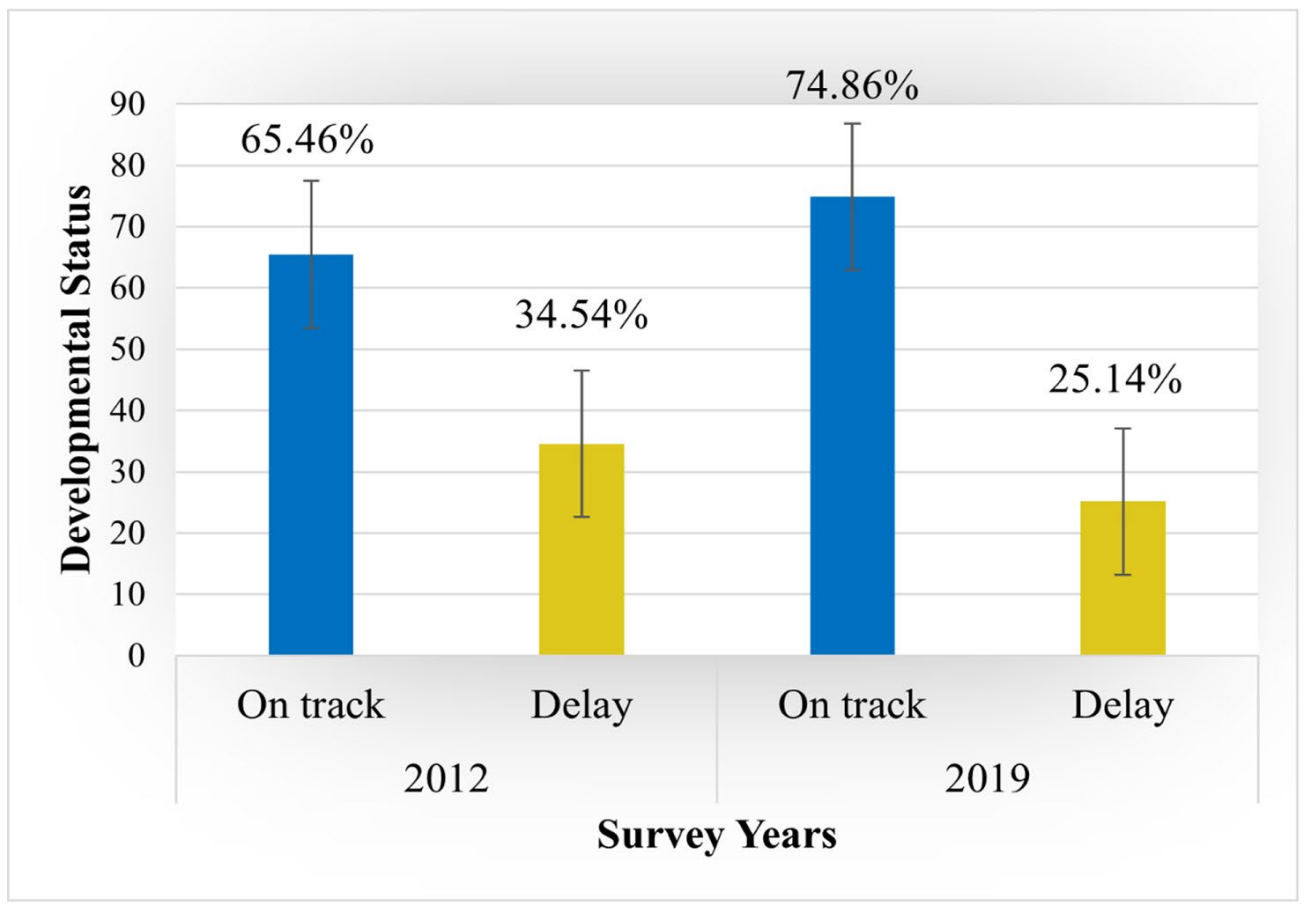
Distribution of developmental status of children by different survey years

The prevalence of developmentally on-track children increased from 65.46% in 2012 to 74.86% in 2019. The change is significant in the proportional test (p < 0.001) (*see* Fig. 2). For two consecutive surveys, the represented sample of child aged 3 was 49.59% in MICS 2012, and 50.82% in MICS 2019, and those aged 4 were 50.41% in MICS 2012 and 49.18% in MICS 2019, respectively. 51.96% of the child in MICS 2012 and 51.60% of the child in MICS 2019 were male, and 48.04% of the child in MICS 2012 and 48.40% of the child in MICS 2019 were female, respectively. Based on residence status, 84.13%of the respondent child in MICS 2012 and 81.44% of the respondent child in MICS 2019 were from rural areas, while 15.87% of the child in MICS 2012 and 18.56% of the child in MICS 2019 were from urban areas, respectively. The distribution of developmentally on track status of 3 years old children was 59.46% according to 2012 MICS, whereas it increased to 68.72% in 2019 MICS and children of 4 years old were 71.40% on track in 2012 MICS whereas that increased to 81.26% in 2019 MICS, respectively. By the sex of the child, the female child was always more developmentally on track than the male child. In 2012 MICS, developmentally on-track status for male children was 63.41% and 67.65% for females. Similarly, in 2019 MICS, developmentally on-track status for male and female children was 71.51% and 78.46%. The children from rural were 72.17% in 2012 MICS and 78.15% in 2019 MICS, who were more developmentally on track than the urban, 63.72% in 2012 MICS and 73.99% in 2019 MICS children respectively [Table 2].

The comparison of ECD on-track status for indicated domains between 2012 and 2019 was assessed (*see* Table 2). The prevalence of this status has increased for each of the domains. The highest increase rate in ECD on-track status (21.2–28.8%) was found in the child literacy-numeracy domain. The lowest rate of increase in ECD on-track status (68.4–72.7%) was found in the child’s social-emotional domain.

**Table 2:** Comparison of the early childhood developmental status, MICS 2012 and 2019 (weighted frequency and percentage)

Characteristics	MICS 2012			MICS 2019		
	Developmentally on track		P-value	Developmentally on track		P-value
	Yes	No		Yes	No	
	**N (%)**	**N (%)**		**N (%)**	**N (%)**	
**Age of child (in years)**						
3	2392 (59.46)	1649(40.54)	< 0.001	3166 (68.72)	1584 (31.28)	< 0.001
4	2909 (71.40)	1198 (28.60)		3680 (81.26)	916 (18.74)	
**Child’s sex**						
Male	2669 (63.41)	1565 (36.59)	0.002	3383 (71.51)	1440 (28.49)	< 0.001
Female	2632 (67.65)	1282 (32.35)		3463 (78.46)	1060 (21.54)	
**Place of residence**						
Urban	4388(63.72)	2467 (36.28)	< 0.001	5541 (73.99)	2070 (26.01)	< 0.001
Rural	913 (72.17)	380 (27.83)		1305 (78.15)	430 (21.85)	
**Division**						
Barishal	526 (67.86)	262 (32.14)	< 0.001	552 (67.8)	269 (32.20)	< 0.001
Chattogram	940 (55.04)	682 (44.96)		1479 (78.26)	470 (21.74)	
Dhaka	1286 (67.45)	674 (32.55)		1453 (81.85)	343 (18.15)	
Khulna	740 (71.70)	326 (28.30)		895 (73.07)	409 (26.93)	
Mymensingh	-	-		347 (61.26)	209 (38.74)	
Rajshahi	527 (66.76)	263 (33.24)		720 (69.57)	307 (30.43)	
Rangpur	866 (78.38)	262 (21.62)		896 (83.71)	207 (16.29)	
Sylhet	416 (54.15)	378 (45.85)		504 (61.73)	286 (38.27)	
**Mother’s Education**						
Primary incomplete	2076 (58.80)	1462 (41.20)	< 0.001	847 (68.56)	389 (31.44)	< 0.001
Primary complete	772 (62.73)	447 (37.27)		1590 (69.38)	727 (30.62)	
Secondary incomplete	1800 (70.01)	760 (29.99)		3363 (76.88)	1143 (23.12)	
Secondary complete or Higher	653 (79.46)	178 (20.54)		1046 (83.11)	241(16.89)	
**Wealth Index**						
Poorest	2621 (60.36)	1696 (39.64)	< 0.001	3026 (69.84)	1331 (30.16)	< 0.001
Middle	1839 (66.06)	886 (33.94)		2574 (75.65)	904 (24.35)	
Richest	841 (77.55)	265 (22.45)		1246 (84.05)	264 (15.95)	
**Religion**						
Islam	4486 (66.08)	2384 (33.92)	0.044	5518 (74.97)	2025 (25.03)	0.658
Others	534 (61.42)	321 (38.58)		908(74.26)	325(25.74)	
**Household Head’s Sex**						
Male	4411 (65.67)	2348 (34.33)	0.852	5500 (75.12)	1996 (24.888)	0.254
Female	609 (65.26)	357 (34.74)		926 (73.42)	354 (26.58)	
**Ethnicity of the household head**						
Bengali	4865 (65.58)	2620 (34.42)	0.798	6684 (74.89)	2438 (25.11)	0.474
Others	155 (66.60)	85 (33.40)		162 (72.70)	62 (27.30)	
**Mother’s Age at the Survey Time**						
15–19	190 (68.00)	86 (32.00)	0.010	1276 (77.02)	428 (22.98)	0.027
20–34	2991 (68.18)	1444 (31.82)		3035 (73.85)	1148 (26.15)	
35+	883 (62.75)	536 (37.25)		2009 (74.83)	751 (25.17)	
**Early Childhood Diseases**						
Yes	1262 (64.63)	712 (35.37)	0.494	1895 (73.84)	738 (26.16)	0.205
No	4035 (65.72)	2132 (34.28)		4940 (75.24)	1761 (24.76)	
**Underweight**						
Yes	1660 (61.12)	1047 (38.88)	< 0.001	1591 (71.86)	667 (28.14)	< 0.001
No	3308 (68.11)	1618 (31.89)		5038 (75.97)	1732 (24.03)	
**Stunned**						
Yes	2037 (59.86)	1355 (40.14)	< 0.001	1749 (70.34)	802 (29.66)	< 0.001
No	2860 (70.83)	1240 (29.17)		4824 (76.79)	1572 (23.21)	
**Wasted**						
Yes	444 (65.59)	239 (34.41)	0.989	647 (74.34)	238 (25.66)	0.721
No	4516 (65.63)	2415 (34.37)		5905 (74.96)	2134 (25.04)	
**Overweight**						
Yes	407 (64.98)	219 (35.02)	0.832	395 (76.0)	158 (24.0)	0.544
No	4894 (65.50)	2628 (34.50)		6451 (74.78)	2342 (25.22)	
**Sanitation**						
Improved	4819 (65.17)	2575 (34.83)	0.348	6218 (74.83)	2279 (25.17)	0.712
Unimproved	201 (61.09)	128 (38.91)		207 (76.0)	71 (24.0)	
**Early childhood education programs**						
Yes	936 (78.93)	246 (21.07)	< 0.001	1498 (85.99)	269 (14.01)	< 0.001
No	4364 (63.31)	2599 (36.69)		5348 (72.19)	2231 (27.81)	
**Mother Stimulation**						
Yes	4619 (66.26)	2422 (33.74)	0.002	5696 (75.86)	1949 (24.14)	< 0.001
No	682 (60.2)	425 (39.8)		1150 (70.2)	551 (29.8)	
**Father Stimulation**						
Yes	3035 (66.49)	1613 (33.51)	0.078	3024 (73.11)	1151 (26.89)	< 0.001
No	2266 (64.02)	1234 (35.98)		3822 (76.29)	1349 (23.71)	
**Other Stimulation**						
Yes	4356 (66.5)	2282 (33.5)	0.003	3646 (74.15)	1344 (25.85)	0.118
No	945 (61.19)	565 (38.81)		3200 (75.68)	1156 (24.32)	
**Inadequate Supervision**						
Yes	542 (62.96)	306 (37.04)	0.343	590 (68.92)	279 (31.08)	< 0.001
No	4754 (65.74)	2538 (34.26)		6256 (75.41)	2221(24.59)	
**Salt Iodization**						
Yes	3600 (64.441)	2075 (35.59)	0.004	5172 (74.65)	1894 (25.35)	0.400
No	1420 (69.03)	629 (30.97)		1251 (75.77)	456 (24.23)	
**Child education Book at home**						
Yes	3325 (72.13)	1251 (27.87)	< 0.001	3837 (80.65)	1017 (19.35)	< 0.001
No	1976 (55.8)	1596 (44.2)		3008 (68.58)	1483 (31.42)	
**Toys**						
Yes	4084 (68.18)	2053 (31.82)	< 0.001	5645 (74.69)	2081 (25.31)	0.465
No	1217 (57.41)	794 (42.59)		1201 (75.67)	419 (24.33)	
**Mass Media**						
Yes	2038 (71.38)	838 (28.62)	< 0.001	3830 (74.69)	1411 (25.31)	0.803
No	2024 (61.88)	1228 (38.12)		2490 (74.95)	916 (25.05)	
**Child Punishment**						
Yes	137 (49.01)	145 (50.99)	< 0.001	321 (64.8)	165 (35.2)	< 0.001
No	5164 (66.13)	2702 (33.87)		6525(75.42)	2335 (24.58)	

The Overall ECD status by their socio-demographic and child characteristics for 2012 MICS and 2019 MICS surveys were shown in Table 3. The distribution of developmentally on track status of child bought in the family of the highly educated mother (Secondary complete or Higher) was 79.46%, according to 2012 MICS, it increased to 81.27% in 2019 MICS. A child bought by a primary incomplete mother was the lowest (58.80%) on track status in 2012 MICS whereas that increased to 68.53% in 2019 MICS still lower than other education groups. By the wealth index of the child family, the richest family’s child was always more developmentally on track than the middle or poorest family’s child. According to the 2012 MICS, the richest family’s child was 77.55% developmentally on track, while the poorest family’s child was 60.36%.

**Table 3:** Factors associated with the developmental status of children, MICS 2012 and 2019

Characteristics	MICS- 2012		MICS- 2019	
	Multivariable		Multivariable	
	Adjusted OR (95% CI)	P-value	Adjusted OR (95% CI)	P-value
**Age of Child**				
4	1.61 (1.39–1.87)	< 0.001	1.78 (1.58-2.00)	< 0.001
3	Reference	-	Reference	-
**Child’s Sex**				
Female	1.42 (1.23–1.64)	< 0.001	1.44 (1.28–1.61)	< 0.001
Male	Reference	-	Reference	-
**Place of residence**				
Rural	1.10 (0.88–1.36)	0.409	0.96 (0.82–1.12)	0.626
Urban	Reference	--	Reference	-
**Division**				
Chattogram	0.59 (0.45–0.76)	< 0.001	1.55 (1.25–1.91)	< 0.001
Dhaka	0.99 (0.77–1.27)	0.924	1.99 (1.61–2.47)	< 0.001
Khulna	1.02 (0.78–1.33)	0.838	1.07 (0.86–1.32)	0.556
Mymensingh	-	-	0.85 (0.65–1.12)	0.242
Rajshahi	0.87 (0.64–1.17)	0.357	0.98 (0.78–1.24)	0.901
Rangpur	1.87 (1.41–2.48)	< 0.001	2.72 (2.17–3.42)	< 0.001
Sylhet	0.62 (0.46–0.85)	0.003	0.83 (0.65–1.07)	0.147
Barishal	Reference		Reference	-
**Mother’s Education**				
Secondary complete or Higher	1.77 (1.29–2.44)	< 0.001	1.50 (1.16–1.93)	0.002
Secondary incomplete	1.25 (1.03–1.51)	0.022	1.21 (1.01–1.45)	0.044
Primary complete	0.97 (0.78–1.22)	0.810	0.96 (0.80–1.16)	0.673
Primary incomplete	Reference	-	Reference	-
**Wealth Index**				
Richest	1.32 (0.98–1.78)	0.067	1.26 (1.08–1.48)	0.003
Middle	1.02 (0.87–1.21)	0.812	1.12 (0.96–1.31)	0.137
Poorest	Reference	-	Reference	-
**Religion**				
Others	1.01 (0.77–1.34)	0.921	--	--
Islam	Reference	-	--	--
**Mother’s Age**				
15–19	1.21 (0.82–1.78)	0.342	1.15 (0.97–1.36)	0.102
20–34	1.15 (0.96–1.38)	0.130	0.95 (0.84–1.08)	0.452
35+	Reference	-		
**Underweight**				
No	1.12 (0.93–1.35)	0.234	1.02 (0.88–1.18)	0.797
Yes	Reference	-	Reference	-
**Stunned**				
No	1.17 (0.98–1.40)	0.082	1.09 (0.95–1.25)	0.215
Yes	Reference	-	Reference	-
			-	-
			-	-
**Early childhood education programs**				
Yes	1.45 (1.14–1.83)	0.002	1.58 (1.32–1.89)	< 0.001
No	Reference	-	Reference	-
**Mother Stimulation**				
Yes	0.96 (0.76–1.22)	0.745	1.01 (0.87–1.19)	0.862
No	Reference	-	Reference	-
**Father Stimulation**				
Yes	-	-	0.78 (0.69–0.87)	< 0.001
No	-	-	Reference	-
**Others Stimulation**				
Yes	1.33 (1.09–1.61)	0.004	-	-
No	Reference	-	-	-
**Inadequate Supervision**				
No	-	-	1.29 (1.07–1.56)	0.009
Yes	-	-	Reference	-
**Salt Iodization**				
No	0.99 (0.84–1.17)	0.928	-	-
Yes	Reference	-	-	-
**Child education Book at home**				
Yes	1.50 (1.27–1.77)	< 0.001	1.54 (1.36–1.75)	< 0.001
No	Reference	--	Reference	-
**Toys**				
Yes	1.54 (1.29–1.85)	< 0.001	-	-
No	Reference	--	-	--
**Mass Media**				
Yes	1.13 (0.95–1.34)	0.180	-	-
No	Reference	--	-	--
**Child Punishment**				
Yes	0.70 (0.49–0.99)	0.043	0.64 (0.50–0.82)	< 0.001
No	Reference	--	Reference	--

Similarly, in 2019 MICS, 84.05% and 69.48% were developmentally on track status for richest and poorest, respectively. The children who were not underweight and not stunned are 68.11% and 70.83% in 2012 MICS were more developmentally on track than the children with malnourished and stunned, respectively. The children who were not underweight and not stunned were 75.97% and 76.79% in 2019 MICS were more developmentally on track respectively than those underweight and stunned. The distribution of developmentally on track status of a child who attends early childhood programs was 78.93%, according to 2012 MICS whereas, it increased to 85.99% in 2019 MICS. A child born into a family where books for children were present had a 72.13% developmentally on track status in 2012 MICS, which increased to 80.65% in the 2019 MICS. In the 2012 MICS, the child born in the family where they experienced punishment were 49.01% developmentally on track, but this increased to 64.80% in the 2019 MICS.

The bivariable and multivariable logistic regression model results refer to the relationship between early childhood development status and children’s socio-demographic profiles in Table 1. To show associations between early childhood developmentally on track status and child age, child sex, geographic location, division, education of mother, household’s wealth index, religion, sex of household head, ethnicity, mother’s age, early childhood diseases, underweight, stunned, wasted, overweight, sanitation, early childhood programs, mother stimulation, father stimulation, other stimulation, inadequate supervision, salt iodization, books, toys, mass media and child punishment. The unadjusted Odds ratio indicates the individual associated with the ECD status. Eighteen variables showed a significant association at the 5% level of significance among all predictor variables (child age, child sex, place of residence, division, mothers education, wealth index, religion, mother age at survey time, underweight, stunned, early childhood education program, mother stimulation, other stimulation, salt iodization, child books, toys, mass media and child punishment) in 2012 MICS and 15 variables showed significant association at 5% level of significance (child age, child sex, place of residence, division, mothers education, wealth index, mother age at survey time, underweight, stunned, early childhood education program, mother stimulation, father stimulation, inadequate supervision, child books and child punishment) in 2019 MICS data.

Children aged 4 were 63% (2012 MICS OR: 1.63, 95% CI: 1.40–1.89) and 78% (2019 MICS OR: 1.78, 95% CI: 1.58-2.00] more developmentally on track than children aged 3. According to child sex, when all other variables were adjusted, the female child had 1.42 times higher (2012 MICS OR: 1.42, 95% CI: 1.23–1.64), and 1.44 times (2019 MICS OR: 1.44, 95% CI: 1.28–1.61) higher developmentally on track status than the male child respectively in both datasets. By comparing both model the odds ratio of division gives different results in some categories. However, Child from Rajshahi [(2012 MICS OR: 0.88, 95% CI: 0.64–1.17) and (2019 MICS OR: 0.98, 95% CI: 0.78–1.24)] and Sylhet [(2012 MICS OR: 0.62, 95% CI: 0.46–0.85) and (2019 MICS OR: 0.83, 95% CI: 0.65–1.07)] division had lower early childhood developmentally on track than Barisal division. In Both surveys, child from Rangpur division had a higher [(2012 MICS OR: 1.87, 95% CI: 1.41–2.48) and (2019 MICS OR: 2.72, 95% CI: 2.17–3.42)] developmentally on track than the Barisal division, respectively. In both multivariable models, children bought by secondary complete or higher educated mothers had a 77% higher (2012 MICS OR: 1.77, 95% CI: 1.29–2.44) and a 50% higher (2019 MICS OR: 1.50, 95% CI: 1.16–1.93) developmentally on track compared to child from primary incomplete or uneducated mothers. In both surveys, the child growing up in rich families were found with 32% higher [OR: 1.32, 95% CI: 0.98–1.78] in MICS 2012 and 26% higher [OR: 1.26, 95% CI: 1.08–1.48] in MICS 2019, developmentally on track than the low-income family. Early childhood education programs play a positive role in early childhood development. In two surveys, the child who attended an early childhood education program were found to have 45% higher [OR: 1.45, 95% CI: 1.14–1.83] in MICS 2012 and 58% higher [OR: 1.58, 95% CI: 1.32–1.89] in MICS 2019, developmentally on track than the children who did not attend in early childhood education program.

Similarly, children who receive father and other stimulation have significantly different ECD statuses. Children with fathers and additional inspiration had a higher status of developmentally on track.

Supervision also plays a crucial role in ECD; compared with inadequate supervision, children raised with adequate supervision were found to have a higher developmentally on track status. Children raised with adequate supervision had a 29% higher developmentally on track. There was a substantial increase in ECD on-track status among the children with books and toys. Similarly, access to mass media by a child’s mother or caretaker significantly impacts early childhood development.

**Fig. 3:**
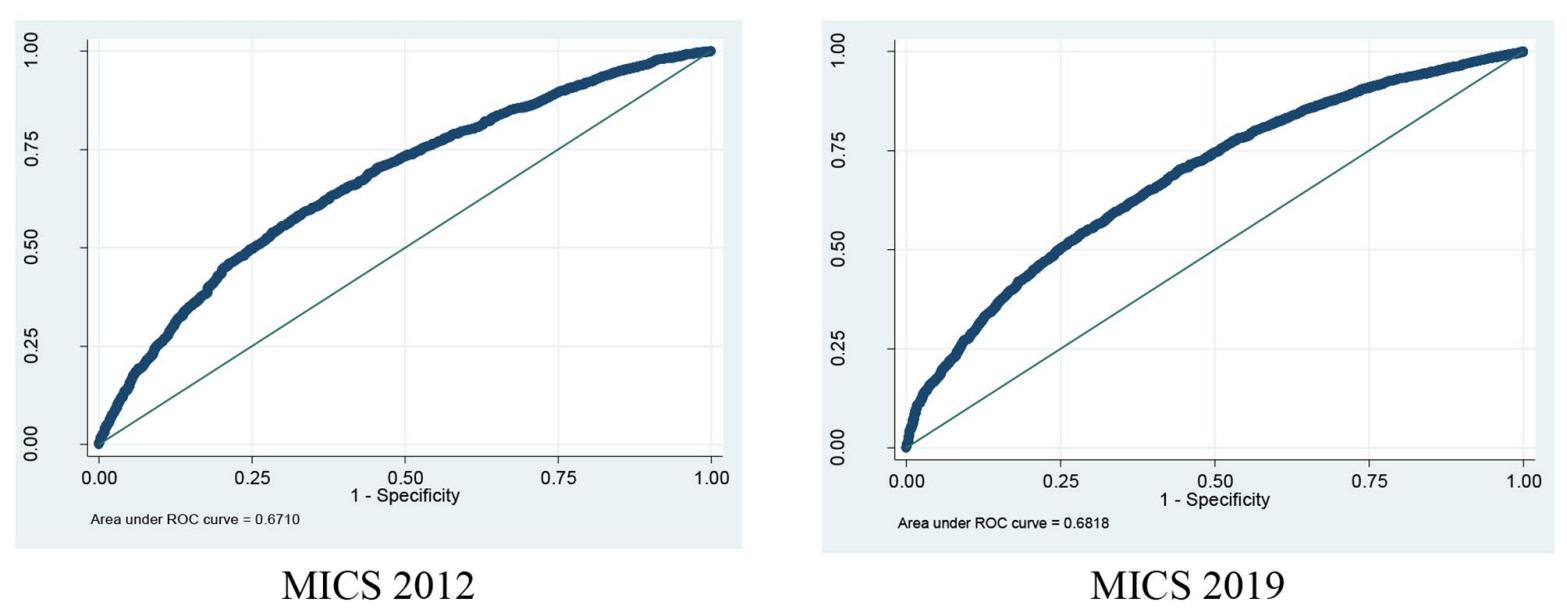
Area Under ROC curve of adjusted model

The intraclass correlation coefficient (ICC) revealed that 10.05% and 7.70% in 2012 and 2019, respectively, of the variation in child ECD status, could be attributed to the difference in the composition of the communities. This also implies that adding the regional characteristics enhanced the model’s capacity to explain differences in childhood ECD status between regions in MICS-2012 than MICS-2019. The table further shows the model fit statistics. The lower AIC, BIC, and Log-likelihood values indicate a better fitting the model. In MICS-2012, the AIC estimates showed a lower value (6828.166) than the MICS-2019 (9432.097). Both surveys also found similar results in BIC (7014.218 Vs 9616.643) (Table 4). For two survey data sets, the areas under the AUROC curve were 0.67 (95% CI: 0.6564–0.6857) in MICS 2012, and 0.68 (95% CI: 0.6695 − 0.6941) in MICS 2019, respectively (*see* Fig. 3). These values indicate that both models were well fitted and could differentiate between the two groups of child development. So, without any doubt, the Multilevel model is better suited for both surveys (*Table S2*).

**Table 4:** Goodness of fit of multivariable logistic regression model, MICS 2012 and 2019

	MICS-2012	MICS-2019
**Cluster level variance (SE)**	0.38 (0.09)	0.27 (0.05)
**ICC (%)**	10.05	7.70
**AIC**	6828.166	9432.097
**BIC**	7014.218	9616.643
**Log-likelihood**	-3386.083	-4690.0483
**Observations**	5,680	8,937

## Discussion

In this study, we used two successive MICS data to investigate the prevalence of ECD status and its associated factors in Bangladesh. We found the prevalence of overall and individual domains of ECD status improved from 2012 to 2019 and the change was statistically significant. The child literacy-numeracy domain and the child social-emotional domain had the highest and lowest rates of ECD on-track status, respectively. This ECDI percentage was lower in Pakistan (Balochistan) and higher in Vietnam [29]. Furthermore, among fifty low- and middle-income nations, the ratio of ECDI fluctuates between the mean rate of children aged 36–59 months on track for child development ranging from 42.6% in Sierra Leone to 85.9% in Belize [29]. We also observed that child age, child sex, residence, division, mother’s education, and wealth index have significant impact on the ECD status.

We observed that the boy’s ECD on-track status was low compared to girls and had a higher chance of developmental delay. These findings were consistent with other studies in Western Cape, South Africa, exploring cognitive, language, and satisfactory motor developmental performance in young children. Emerson et al. also showed that boys with developmental delays were more prevalent in Bangladesh, Pakistan, and Vietnam [30].

Compared to their poorer counterparts, children from the wealthiest families had a better chance of overall development. Moreover, children from the second-lowest socioeconomic status have poorer health and development than children from the highest socioeconomic status. Emerson et al. showed their study that in five of the six countries, children with developmental delays were more likely to live in poverty than their peers. In three countries, the differences were statistically significant (Bangladesh, Laos, and Vietnam). Vietnam had the highest relative disadvantage rates, with children with developmental delays being 2.2 times more likely to be poor [31]. Another study noted that poverty and traumatic childhood experiences affect brain development and cognition in long-term physiological and epigenetic ways [32].

Malnutrition caused developmental delays in children, according to our research. Black et al. also mentioned that children who were malnourished or frequently ill were more likely to experience developmental problems [33]. Therefore, highlighting the necessity of implementing coordinated early childhood development programs in collaboration with the health and nutrition sectors.

Early childhood programs were essential to support young children’s mental and physical development [34]. According to our study findings, children who attended an early childhood education program were much more developmentally on track than their peers.

Adequate supervision, stimulation, and having books and toys in the household positively impacted ECD on track status. Noble et al. [35] showed that nurturing care has been linked to children’s health, growth, and development worldwide, and neuroscientific data suggests that enabling care during early childhood mitigates the negative impacts of poor socioeconomic position on brain development.

In our study, access to mass media by the home or caregivers increased the likelihood of early childhood developmentally on track status. Television and other forms of media could make it easier for children and parents to access early childhood development programming at home [33]. Sesame Street was a children’s educational television program broadcast over 150 countries [36]. Nearly half of Bangladesh’s 3-5-year-old children watch television every day, with 83% of urban and 58% of rural pre-schoolers watching Sesame Street [37]. According to a meta-analysis of over 10,000 children from 15 nations, watching Sesame Street increases reading and numeracy domains, health and safety, social reasoning, and attitudes toward others [38].

Child punishment has been linked to developmental delays in children. Physical abuse, family instability, risky neighbourhoods, and poverty can all lead to youngsters with poor coping skills, difficulties regulating emotions, and lower social functioning than their peers [38].

### Strengths and limitations

To the best of our knowledge, this is the first study with Bangladeshi children using the most recent MICS data to assess developmental status using ECDI scores. We utilized a big enough nationwide survey dataset to represent the whole Bangladeshi population.

Despite these strengths, our study had several limitations. Because we used secondary data, we had no control over the variables we chose, the data quality, or the measurement indicators. Only data on child development for children aged three and four are available. It’s unclear how younger children’s developmental ratings compare to those of 3- and 4-year-olds. More data from birth to five years is needed to better understand children’s development at the national level. In addition, the survey was conducted in 2012 and 2019. As a result, the developmental status may have shifted since then.

### Recommendations

The study’s conclusions should be taken into account by governments, international agencies, non-governmental organizations, and public health professionals striving to improve early childhood development. According to ECD, further research is needed to produce more detailed and age-specific assessments that can better capture children’s abilities across a range of cultures and local contexts. Beyond the conventional developmental criteria, additional work is required to understand the special needs of children who may have more serious difficulties requiring more intense therapy and care. In response to the loss of human potential associated with early adversity, leaders from global organizations have issued urgent demands for solutions to ensure that young children realize their developmental potential. Storytelling, singing, and playing with household objects are all low-cost activities that provide early development experiences for young children.

## Conclusion

The study focuses on the level and influencing factors of early childhood developmental status among Bangladeshi children aged 3 and 4 years. In Bangladesh, many children who are developmentally on track come from higher socioeconomic status families, while the poorest children are mostly experiencing developmental delays. Other important factors influencing ECD status revealed by our research include early childhood education programs, families’ possession of children’s books, mothers’ educational level, and wealth index. The findings of our study will aid public health initiatives in Bangladesh in improving their ECD programs.

## Electronic supplementary material

Below is the link to the electronic supplementary material.

Supplementary Material 1

## Data Availability

Data from the 2012 and 2019 Bangladesh MICS (Multiple Indicator Cluster Surveys) were used in this study, which was based on secondary analysis (https://mics.unicef.org/surveys).
