# Supplementary material for: Early childhood developmental status and its associated factors in Bangladesh: a comparison of two consecutive nationally representative surveys
Source: BMC Public Health. 2023 Apr 12;23:687. doi: 10.1186/s12889-023-15617-8 (PMC10099688; doi:10.1186/s12889-023-15617-8)
Supplement: Supplementary file 1 — Supplementary Material 1 [file 12889_2023_15617_MOESM1_ESM.docx]

**Table S1** Factors associated with the developmental status of children, MICS 2012 and 2019.

| **Characteristics** | **MICS- 2012** | | **MICS- 2019** | |
| --- | --- | --- | --- | --- |
|  | **Univariate** | | **Univariate** | |
|  | **Unadjusted**  **OR (95% CI)** | **P-value** | **Unadjusted**  **OR (95% CI)** | **P-value** |
| **Age of Child** | | | | |
| 4 | 1.70 (1.52-1.90) | <0.001 | 1.97 (1.77-2.20) | <0.001 |
| 3 | Reference | - | Reference | - |
| **Child’s Sex** | | | | |
| Female | 1.21 (1.07-1.36) | 0.002 | 1.45 (1.31-1.61) | <0.001 |
| Male | Reference | - | Reference | - |
| **Place of residence** | | | | |
| Rural | 1.48 (1.21-1.80) | <0.001 | 1.26 (1.08-1.46) | 0.003 |
| Urban | Reference | - | Reference | - |
| **Division** | | | | |
| Chattogram | 0.58 (0.47-0.72) | <0.001 | 1.71 (1.41 - 2.08) | <0.001 |
| Dhaka | 0.98 (0.79-1.22) | 0.867 | 2.14 (1.76 -2.61) | <0.001 |
| Khulna | 1.20 (0.95-1.51) | 0.119 | 1.29 (1.06-1.57) | 0.011 |
| Mymensingh | - | - | 0.75 (0.59 - 0.96) | 0.023 |
| Rajshahi | 0.95 (0.75-1.21) | 0.686 | 1.09 (0.88-1.34) | 0.445 |
| Rangpur | 1.72 (1.37-2.16) | <0.001 | 2.44 (1.97-3.02) | <0.001 |
| Sylhet | 0.56 (0.44-0.71) | <0.001 | 0.77 (0.61-0.96) | 0.020 |
| Barishal | Reference | - | Reference | - |
| **Mother’s Education** | | | | |
| Secondary complete or Higher | 2.71 (2.14-3.43) | <0.001 | 2.26 (1.82-2.80) | <0.001 |
| Secondary incomplete | 1.64 (1.43-1.87) | <0.001 | 1.53 (1.31-1.78) | <0.001 |
| Primary complete | 1.18 (0.99-1.40) | 0.061 | 1.04 (0.88-1.23) | 0.651 |
| Primary incomplete | Reference | - | Reference | - |
| **Wealth Index** | | | | |
| Richest | 2.27 (1.84-2.79) | <0.001 | 1.73 (1.53-1.96) | <0.001 |
| Middle | 1.28 (1.13-1.44) | <0.001 | 1.33 (1.15-1.53) | <0.001 |
| Poorest | Reference | - | Reference | - |
| **Religion** | | | | |
| Islam | 0.95 (0.77-1.17) | 0.652 | 1.04 (0.88-1.23) | 0.666 |
| Others | Reference | - | Reference |  |
| **Household’s Head Sex** | | | | |
| Male | 1.01 (0.77-1.31) | 0.960 | 1.09 (0.94-1.27) | 0.255 |
| Female | Reference | - | Reference |  |
| **Ethnicity** | | | | |
| Bengali | 0.89 (0.64-1.25) | 0.512 | 1.12 (0.82-1.53) | 0.473 |
| Others | Reference | - | Reference |  |
| **Mother’s Age** | | | | |
| 15 – 19 | 1.26 (0.90-1.77) | 0.182 | 1.13 (0.97-1.31) | 0.121 |
| 20 – 34 | 1.27 (1.09-1.49) | 0.003 | 0.95 (0.84-1.07) | 0.404 |
| 35+ | Reference | - | Reference | --- |
| **Early Childhood Diseases** | | | | |
| No | 1.05 (0.91-120) | 0.497 | 1.08 (0.96-1.21) | 0.208 |
| Yes | Reference | - | Reference |  |
| **Underweight** | | | | |
| No | 1.36 (1.19-1.55) | <0.001 | 1.24 (1.10-1.39) | < 0.001 |
| Yes | Reference | - | Reference | - |
| **Stunned** | | | | |
| No | 1.63 (1.44-1.84) | <0.001 | 1.40 (1.25-1.56) | < 0.001 |
| Yes | Reference | - | Reference | - |
| **Wasted** | | | | |
| No | 1.01 (0.81-1.23) | 0.989 | 1.03 (0.86-1.23) | 0.721 |
| Yes | Reference | - | Reference |  |
| **Overweight** | | | | |
| Yes | 0.98 (0.79-1.21) | 0.831 | 1.07 (0.86-1.32) | 0.543 |
| No | Reference | - | Reference |  |
| **Sanitation facility** | | | | |
| Unimproved | 0.75 (0.64-0.88) | <0.001 | 1.06 (0.76-1.48) | 0.711 |
| Improved | Reference | - | Reference | - |
| **Early childhood education programs** | | | | |
| Yes | 2.17 (1.79-2.64) | <0.001 | 2.37 (2.00-2.79) | < 0.001 |
| No | Reference | - | Reference | - |
| **Mother Stimulation** | | | | |
| Yes | 1.30 (1.10-1.53) | 0.002 | 1.33 (1.17-1.52) | < 0.001 |
| No | Reference | - | Reference | - |
| **Father Stimulation** | | | | |
| Yes | 1.12 (0.99-1.26) | 0.083 | 0.84 (0.76-0.94) | 0.002 |
| No | Reference | - | Reference | - |
| **Others Stimulation** | | | | |
| Yes | 1.26 (1.08-1.47) | 0.003 | 0.92 (0.83-1.02) | 0.125 |
| No | Reference | - | Reference | - |
| **Inadequate Supervision** | | | | |
| No | 1.13 (0.87-1.46) | 0.355 | 1.38 (1.16-1.65) | <0.001 |
| Yes | Reference | - | Reference | - |
| **Salt Iodization** | | | | |
| No | 1.26 (1.10-1.43) | <0.001 | 1.06 (0.92-1.22) | 0.406 |
| Yes | Reference | - | Reference | - |
| **Child education Book at home** | | | | |
| Yes | 2.05 (1.83-2.30) | <0.001 | 1.91 (1.71-2.13) | <0.001 |
| No | Reference | - | Reference | - |
| **Toys** | | | | |
| Yes | 1.59 (1.37-1.85) | <0.001 | 1.05 (0.91-1.22) | 0.479 |
| No | Reference | - | Reference | - |
| **Mass Media** | | | | |
| Yes | 1.54 (1.34-1.77) | <0 .001 | 0.99 (0.88-1.10) | 0.807 |
| No | Reference | - | Reference | - |
| **Child Punishment** | | | | |
| Yes | 0.49 (0.36-0.67) | <0.001 | 0.60 (0.49-0.74) | <0.001 |
| No | Reference | - | Reference | - |

Table S2: Area Under ROC curve of adjusted (final) model for both MICS 2012 and 2019

| Survey Year | Area Under ROC Curve | |
| --- | --- | --- |
|  | AUC (95% Confidence Interval) | P-value |
| MICS 2012 | 0.6710 (0.6564 – 0.6857) | <0.001 |
| MICS 2019 | 0.6818 (0.66953 - 0.69406 | <0.001 |
